# Evaluating Reach, Acceptability, Utility, and Engagement with An App-Based Intervention to Improve Medication Adherence in Patients with Coronary Heart Disease in the MedApp-CHD Study: A Mixed-Methods Evaluation

**DOI:** 10.3390/medsci7060068

**Published:** 2019-06-04

**Authors:** Karla Santo, Anna Singleton, Clara K Chow, Julie Redfern

**Affiliations:** 1 Westmead Applied Research Centre, Westmead Clinical School, Faculty of Medicine and Health, The University of Sydney, Sydney, NSW 2145, Australia; anna.singleton@sydney.edu.au (A.S.); clara.chow@sydney.edu.au (C.K.C.); Julie.redfern@sydney.edu.au (J.R.); 2 The George Institute for Global Health, Sydney, NSW 2050, Australia; 3 Westmead Hospital, Cardiology Department, Sydney, NSW 2145, Australia

**Keywords:** mixed-methods evaluation, medication adherence, coronary heart disease, mHealth, smartphone, apps

## Abstract

Objective: The aim of this study was to assess the reach, acceptability, utility, and engagement with the apps that were used in the MEDication reminder APPlications (apps) to improve medication adherence in Coronary Heart Disease (MedApp-CHD) study, a randomised clinical trial to improve medication adherence, using a mixed-methods approach. Methods: The MedApp-CHD study randomised 163 patients with coronary heart disease (CHD) to one of three groups: (i) usual care (*n* = 56), (ii) a basic medication reminder app (*n* = 54), or (iii) an advanced medication reminder app (*n* = 53). For this mixed-methods evaluation, the data sources included patient screening logs, feedback questionnaires collected at three-month follow-up, focus groups discussions, and analytical data from the app software. Results: Ninety-four percent (98/104) of participants who received a medication reminder app completed the three-month feedback questionnaire and 15 participated in the focus group discussions. The themes that were identified included that participants (i) found the medication reminders useful in reminding them to take the medications on the correct time every day, (ii) liked having the medication list as an easily-accessible record of medications’ names and dosages, (iii) reported being likely to continue to use the apps after the study completion, (iv) would be likely to recommend the apps to their family and friends, and (v) those who used the clinical measurements feature found it useful as a tool to track and graph the blood pressure and glucose levels over time (especially those with diabetes and/or hypertension). In addition, analytical data from the app software demonstrated that the participants used the medication-related features more than the clinical measurements feature. Furthermore, data from the patient screening logs showed that the main reason for exclusion, other than not meeting the CHD criteria, was not having a suitable smartphone, and those that were excluded for this reason were older and had a higher proportion of females than those enrolled in the study. Conclusion: This study provides important insights regarding the features that are most useful in apps that aim to improve medication adherence. This mixed-methods evaluation suggests that, currently, young male patients with CHD are more likely to use such apps, that the apps were well-accepted and useful in reminding the patients to take the medications, and that the patients were engaged in regularly using the apps.

## 1. Introduction

Together with lifestyle modification, medication adherence is a key component of prevention of coronary heart disease (CHD), which is the leading cause of death globally [1]. Good adherence to medications is associated with lower risk of cardiovascular diseases [2]. However, adherence to cardiovascular medications is sub-optimal worldwide. It was estimated that only around 60% of patients have good adherence to cardiovascular medications [2,3]. To tackle this problem, interventions aimed at improving medication adherence in patients with CHD have been designed, tested and shown to be effective [4].

Recently, given the worldwide exponential growth in mobile phone ownership, mobile technologies have been increasingly used in healthcare. Specifically, a growing body of evidence has investigated the use of mobile technology as a potential tool for improving medication adherence. A recent meta-analysis has shown that text-messages were effective in improving medication adherence in chronic diseases [5]. In addition, recent studies [6,7,8], including the MEDication reminder APPlications (apps) to improve medication adherence in Coronary Heart Disease (MedApp-CHD) study, have shown that the use of smartphone apps is associated with improved adherence to cardiovascular medications.

Although these studies have shown that apps are effective, there is a lack of evidence regarding patients’ acceptability, perceived utility and benefits of using such apps, as well as the potential reach of these app-based interventions. Evaluating these factors is extremely important in informing future development and implementation of app-based interventions in different settings and populations, as well as facilitating intervention scalability. Therefore, the aim of this study was to assess the reach, acceptability, utility and engagement with the apps used in the MedApp-CHD study, using a mixed-methods approach.

## 2. Methods

### 2.1. Study Design

This study is a mixed-methods approach to evaluate the app-based intervention used in the MedApp-CHD study. The study design and the main results have been previously reported [9,10]. In brief, the MedApp-CHD study was a parallel-design, single-centre, single-blind randomized clinical trial (RCT), which aimed to assess whether publicly available high-quality medication reminder apps were effective in improving medication adherence in patients with CHD, compared to usual care. An additional aim of the MedApp-CHD study was to evaluate whether an app with additional features would improve adherence further. The study randomised patients with CHD to receive either usual care, a basic medication reminder app with basic features or an advanced medication reminder app with more interactive and customisable features. The primary outcome of the study was medication adherence at three months, measured by the 8-item Morisky Medication Adherence Scale [11,12,13]. All patients provided written informed consent and ethical approval was obtained from the Western Sydney Local Health Network Human Research Ethics Committee (HREC/1/WMEAD/3).

A mixed-methods evaluation is a research approach, in which the researchers collect and analyse both quantitative and qualitative data within the same study [14]. In this mixed-methods evaluation, we have focused on aspects of intervention content and delivery, such as the different intervention components, acceptability and perceived utility/benefits of the intervention, and reach of the intervention [15]. The specific objectives of this mixed-methods evaluation are presented in Table 1.

### 2.2. Interventions

The medication reminder apps that were used in the study were selected in a systematic review and stepwise process to evaluate the features and quality of all publicly available apps at the Australian iTunes and Google Play stores. This review has been previously published, but, in summary, it classified the apps into basic or advanced medication reminder apps depending on whether they had additional interactive and customisable features [16]. Through a quality assessment process, the highest quality app in each category was selected to be used in the study. Both of the apps were freely available in the app stores. All intervention participants received instructions and assistance to download the app, input the cardiovascular medications into the app, and set daily reminders for medication-taking. In addition, both the basic and the advanced app groups had a non-medication related feature, in which the participants could record and track clinical measurements, such as blood pressure (BP) and glucose levels.

The basic app had a basic feature of providing non-interactive one-time-only daily reminders for medication-taking every day, which was similar to an alarm or text-message. The advanced app had additional interactive and customisable features, including: (1) medication-taking tracking, in which the medication doses could be marked as ‘taken’ or ‘missed’; (2) snooze option and default settings, in which the daily reminders could be snoozed and repeated up to three times at regular intervals or until the medication was marked as ‘taken’; and, (3) other medication-related features, which included weekly percentage of adherence, reminders to refill the medication, option to export and share information with others, and a peer-support feature, in which a family member or friend could be alerted if the participant missed a medication dose.

### 2.3. Data Sources

(1) Recruitment screening logs: A screening log to determine eligibility and reason for non-participation was kept as part of the study recruitment procedures to examine the reach and potential generalisability of the app-based intervention. Information on age and gender was collected for all patients that were screened to participate in the study. The screening log information was stored in a secure electronic database.

(2) Feedback questionnaire: All participants in both intervention groups (basic and advanced medication reminder groups) were invited to answer a feedback questionnaire after the blinded three-month follow-up assessment. The feedback questionnaire comprised a total of 15 items, 11 questions asked about the app’s perceived usefulness and ease-of-use with five-point Likert-scale responses with the following options; strongly agree, agree, neutral, disagree, and strongly disagree; another three questions asked about the frequency of app usage and occurrence of technical problems with five multiple-choice answer options, ranging from daily to never; and one final free-text feedback item provided the opportunity for participants to make any suggestions or other comments about the app. In addition, participants in the advanced medication reminder app group were asked to answer another four Likert-scale questions about the additional features of the advanced app. These four additional questions required five-point Likert-scale responses (strongly agree to strongly disagree) asking about the perceived usefulness of the snooze option, the ability to track ‘taken’ and ‘missed’ doses, the ability to share information with family members and health professional, and additional medication information, which were features only present in the advanced medication reminder app.

(3) Focus groups: At the end of the three-month follow-up interview, all participants in both intervention groups were invited to participate in a focus group discussion to explore their views about the app intervention. Random sampling was used to ensure that a variety of viewpoints were explored, as well as stratified purposeful sampling was used to ensure female representativeness in the groups. The discussions were conducted using standard focus groups methods, which included an experienced facilitator (JR), scribes (KS and AS), the setting of ground-rules and audio recording (two devices). A focus group discussion guide with a set of 16 questions was used to prompt the discussion around the following key aspects of the app-based intervention; (1) the usefulness of the app and specific app features, such as the medication list, the medication reminders, and tracking of clinical measurements; (2) the perceived benefits and potential impact of app use on medication-taking and other health-related behaviours; and (3) engagement with the app by means of frequency of use, ease-of-use vs. difficulties/challenges in using the app, technical issues, and family support in using the app. A few examples of the questions are presented in Table 2. The focus groups discussions took place at the local hospital, where the patients were enrolled in the study.

(4) Analytical data: App background data was directly collected from the app software to evaluate the app usage patterns for the advanced app. The information collected included the number and names of medications entered into the app by the participants and a percentage of adherence, which was calculated by dividing the number of times that the participant marked the medication as ‘taken’ compared to the scheduled medication doses. Information on the number of times that the participants recorded a clinical measurement, such as weight, BP and glucose levels, as well as their values were also collected.

### 2.4. Analysis

Demographic information and the quantitative results were summarised as means and standard deviations (SD) or medians and interquartile ranges (IQR) for the continuous variables, and as frequencies and percentages for the categorical variables. To facilitate the understanding of the results, the responses of five-point Likert-scale questions of the feedback questionnaires were merged into three categories. For example, for questions with responses ‘strongly agree’ to ‘strongly disagree’, the ‘strongly agree’ and ‘agree’ categories were combined to ‘agree’ and the ‘strongly disagree’ and ‘disagree’ categories were combined to ‘disagree’. Data was analysed based on the three remaining categories ‘agree’, ‘neutral’ and ‘disagree’.

The focus group audio-recordings were transcribed verbatim and complemented with the scribes’ notes when the recordings were unclear. One researcher (KS) coded the transcripts, line by line, and developed a coding framework using the constant-comparison method [17]. The coder is an experienced clinician-researcher with expertise in cardiovascular disease management, and quantitative and mixed-methods research. The content was constantly compared with previously coded data throughout the coding process. This process was done until no new concepts were identified. This process also examined the individual participant’s perspective, as well as the emerging consensus between the participants around the issues being discussed. Focus groups participants’ quotes were used to illustrate the key emergent themes. All the analyses were performed using statistical software SPSS version 25 (IBM Corporation, Armonk, NY, USA).

## 3. Results

The MedApp-CHD study main results were previously published [10]. In summary, 163 patients with CHD were randomised to: (i) usual care (*n* = 56), (ii) the basic medication reminder app (*n* = 54) or (iii) the advanced medication reminder app (*n* = 53). At three months, the participants in the medication reminder app groups had a significantly higher medication adherence, when compared to the usual care group. There was no significant difference in adherence between the basic and advanced app groups.

**Figure 1:**
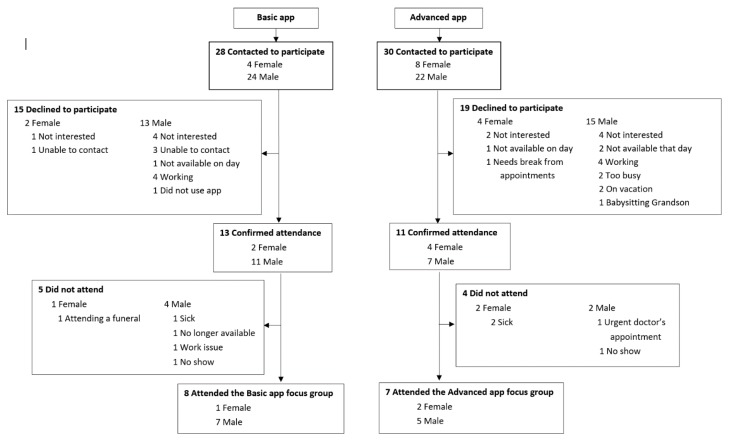
Focus groups participation flowchart.

In this mixed-methods evaluation, we evaluated the recruitment screening logs for the 2308 patients screened to participate in the MedApp-CHD study. In addition, 98 feedback questionnaires were collected and analysed (98/104, 94% response rate); 50 from participants in the basic medication reminder app group and 48 from participants in the advanced medication reminder app group. Two focus groups discussions were held in September 2017 at the Westmead Hospital in Sydney, Australia. Fifty-eight participants were invited to participate in either the basic or the advanced app focus group discussions, with 24 participants accepting the invitation; however, only 15 participants attended these discussions (Figure 1). One focus group (FG 1) was conducted with eight participants who were randomised to the basic medication reminder app, while the other focus group (FG 2) was conducted with seven participants who were randomised to the advanced medication reminder app. The number of male vs female participants and reasons for non-participation are presented in Figure 1.

### 3.1. Reach and Potential Generalisability

As previously reported in the main MedApp-CHD results paper [10], 2308 patients were screened to participate in the study and 2142 were deemed as ineligible. The main reason for ineligibility was the lack of CHD diagnosis (1576/2142, 74%). Among the remaining 566 patients who had CHD, 220 (39%) did not own a smartphone that was suitable to deliver the app intervention, including those who: (1) did not own a mobile phone (29/566, 5%), (2) owned a non-smartphone mobile phone (177/566, 31%), and (3) owned a smartphone that did not operate with either iOS or Android systems (14/566, 2%). The other reasons for exclusion included declining to participate (109/566, 19%), not being proficient in English (97/566, 17%), already using another medication reminder app or alarm reminder (27/566, 5%), and other non-specific reasons (113/566, 20%).

In terms of differences between the excluded patients and the enrolled participants, when comparing the 1576 patient who were excluded for not having CHD with the enrolled participants, those excluded had a younger age and a higher proportion of females (Table 3). In addition, when comparing the 566 patients who had CHD and were still ineligible to participate with the enrolled participants, those that were excluded were significantly older (respectively, mean age 62.9, SD 10.41 years vs. 57.9, SD 8.91 years, *p* < 0.001) and had a significantly higher proportion of females (respectively, 116/566, 20.5% vs. 20/163, 12.3%, *p* = 0.018). Comparisons of age and gender between the enrolled participants and ineligible patients categorised by the specific reasons for non-participation are presented in Table 3. Furthermore, when comparing the sub-categories of the exclusion criteria ‘no suitable smartphone’, there were significant differences in age (*p* = 0.004), but not in terms of gender (*p* = 0.413) between (1) those who did not own a mobile phone (age 65.5, SD 8.11 and 24.1% females), (2) those who owned a non-smartphone mobile phone (age 69.0, SD 10.49 and 20.3% females), and (3) those who owned a smartphone that did not operate with either iOS or Android systems (age 60.6, SD 8.11 and 7.1% females).

### 3.2. Acceptability, Utility and Engagement with the Apps

The baseline characteristics of the 98 feedback questionnaire respondents and the 15 focus group participants are presented in Table 4. These two group of participants had similar characteristics in terms of demographics and medical history.

#### 3.2.1. Feedback Questionnaire Responses

**Figure 2:**
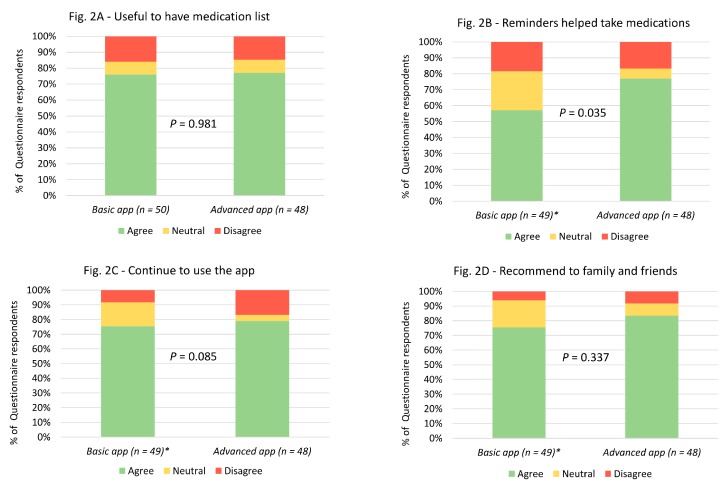
Utility-related questionnaire responses in both basic and advanced app groups. *1 missing response in the basic medication reminder app group.

In terms of overall utility, the majority of the study participants who responded to the feedback questionnaire, in both basic and advanced app groups, found the apps to be useful in terms of having their medication list on their smartphones and reminding them to take their medication at the correct time (Figure 2). In addition, most of the participants said that they would continue to use the app beyond the study completion and would recommend it to a family member or friend (Figure 2). Importantly, there were no significant differences in the responses between the participants in the basic app and the advanced app groups, except that more participants in the advanced app group agreed that the reminders helped them to take their medication, when compared to the basic app group (Figure 2).

**Figure 3:**
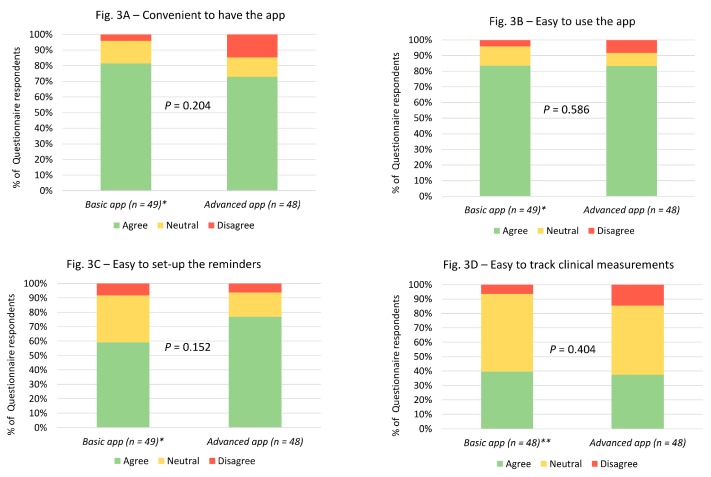
Acceptability-related questionnaire responses in both basic and advanced app groups. * 1 missing response in the basic medication reminder app group. ** 2 missing responses in the basic medication reminder app group.

Looking at acceptability aspects of the app-based intervention, most participants in both basic and advanced app groups found it convenient to have the medication reminder app on their smartphones, found the app easy-to-use, and found it easy to set up the reminders (Figure 3). However, not many patients found it easy to track their clinical measurements in the app (Figure 3). There were no significant differences in the participants’ responses between the two groups.

**Figure 4:**
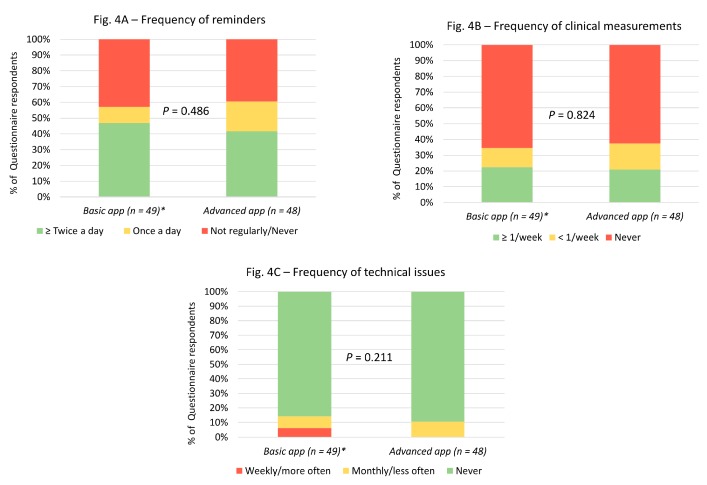
Engagement-related questionnaire responses in both basic and advanced app groups. *1 missing response in the basic medication reminder app group.

Regarding engagement, most patients used the app to remind them to take their medication at least once a day (Figure 4). However, few participants used the feature to record and track clinical measurements (Figure 4). Similarly, not many participants had technical issues while using the app (Figure 4).

**Figure 5:**
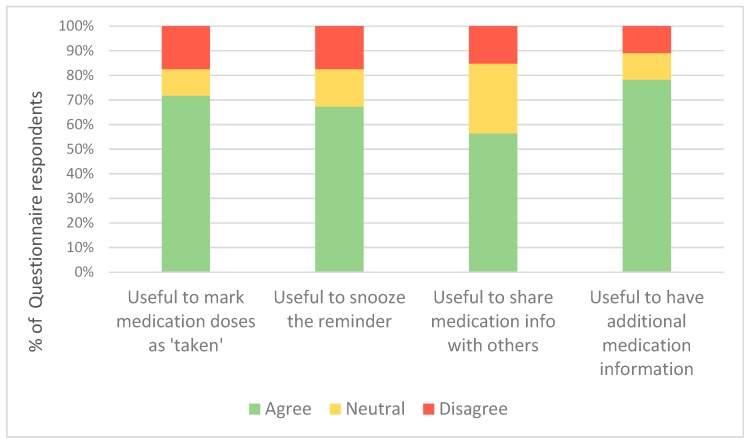
Additional feedback questionnaire responses in the advanced medication reminder app group.

Regarding the additional customisable and interactive features of the advanced app, most feedback questionnaire respondents in this group found it useful to mark the medication dose as ‘taken’, have a snooze option, share the medication information with family and health professionals, and have additional medication information in the app (Figure 5).

#### 3.2.2. Focus Groups Discussions

Overall, the medication reminder apps were considered to be useful to the participants. Some of the themes that were identified in focus groups related to the utility of the apps (Table 5) included that participants (i) found the medication reminders useful in reminding them to take their medication at the correct time every day, (ii) liked having the medication list as an easily-accessible record of medications’ names and dosages, (iii) reported being likely to continue to use the apps after study completion, (iv) would be likely to recommend the apps to their family and friends, and (v) those who used the clinical measurements feature found it very useful as a tool to track and graph blood pressure and glucose levels over time (especially those with diabetes and/or hypertension).

An interesting aspect of the utility of the reminders was that the patients reported that the reminders changed their behaviours, by teaching them a routine in regard to their medication-taking behaviour with illustrative quotes below. Although some of the participants relied on the app reminders to remember to take their medications every day, others used it as a final reminder. These participants reported they would usually take their medications before the reminder alert, but sometimes they would forget and indicated that the app reminder was useful in those situations, especially when their daily routine had changed, for example, when travelling or their prescription was changed.

“I look at it like it’s a conditioned learning. If I end up taking my medicine, you know, 5 minutes before it goes off…It’s teaching me a routine.”

(D, male, 33 years, FG 1)

“When I first put it [the app] on [the smartphone], it was very very helpful…for 3, 4 months…but I got used to it so automatically, I don’t look in the app, automatically I’m doing it and not missing any [medications].”

(L, male, 69 years, FG 2)

“So I had a system where every night my kind wife puts a little box with all the tablets every night and I just use that. But when it changes and like if I have to take a flu medication for a while or something, I found it really useful…I found that really useful to remind me to take the additional tablets”

(R, male, 59 years, FG 2)

Interestingly, the participants mentioned that the app reminders were useful, not only in teaching them a medication-taking behaviour, but the reminders also acted as a trigger for them to take care of their health in general and maintain other healthy habits.

“One thing that’s really good, you’ve got your personal reminder…if you’ve got kids and they’re sick or their regular medication, you’re concentrating on your kids, number 1, and you’re number 2. If you don’t have that reminder, quite possibly by the time you’ve finished with the kids, you’d forget your own medication. So that personal reminder I think is great.”

(T, female, 61 years, FG 1)

“There’s been times I think all of us put other things before ourselves and other things and circumstances come across and up all the time and this was just you know, sort of said, ‘Hey, regardless of that, this has to be done, otherwise’…”

(D, male, 33 years, FG 1)

“If I’m arranging photographs and doing things and just totally preoccupied so when this little thing goes off at 10:30 pm…It also goes ‘Go to bed!’…In a strange way... it does re-educate you and refocus you, you know? Like, I never expected to get that benefit from something external that wasn’t human.”

(D, male, 64 years, FG 1)

In terms of acceptability and engagement, the apps were generally well-accepted and the participants found them easy-to-use, although they did report some difficulties in changing a medication or adding new ones in the app with illustrative quotes, as follows.

“It was straight forward stuff. It was easy”

(B, male, 62 years, FG 1)

“It’s not as intuitive as I liked to see applications, it makes modifications a little bit challenging for me.”

(D, male, 64 years, FG 1)

“I thought it was confusing…to change a medication.”

(M, male, 57 years, FG 2)

Also related to engagement, the participants talked about the importance of having healthcare professionals and family members support in using the app.

“I showed [the app] to the chemist. He said, ‘it’s really good’, I mean, he was very impressed.”

(T, male, 57 years, FG 1)

“They’d call out ‘Mom! That was your medication reminder.’ They’d know, so if I missed it, they’d be telling me.”

(T, female, 61 years, FG 1)

“I think it’s like…you’re getting your kids and your family more, sort of, involved in using that and sort of they become part of that whole network…Yeah, my younger one, every time it goes off he goes ‘Dad! Medication!’ and I say ‘Yeah, I’ve taken it’, you know?”

(J, male, 59 years, FG 1)

Regarding the additional customisable and interactive features that were only present in the advanced medication reminder app, participants in the advanced medication reminder app group found the snooze option and three-time default reminder very useful. Moreover, the participants in the basic medication reminder app group said that they would have liked the basic app to have a snooze option.

“I found I need a snooze. Because sometimes you’re in the middle of something and it only rings the once…and you might be driving, and you think okay so when I get home…but you forget by the time that happens.”

(T, female, 61 years, FG 1)

In the advanced app focus group discussion, participants also mentioned that they found marking the medication dose as ‘taken’ was very useful.

“…it’s good if you sometimes, if you don’t remember whether you’ve taken a certain tablet or not, it helps you ‘Aw, did I take that or didn’t I?’ Yeah, you could have a look [on the app] ‘Aw, yeah, okay, I did take it’.”

(L, female, 54 years, FG 2)

“I find it sort of helps complete the process. Like, I was very cautious and never would click it…until I had physically taken it because I know if I click it…it’s all out of my head.”

(M, male, 57 years, FG 2)

### 3.3. App Usage Patterns (Advanced Medication Reminder App Only)

Of the 52 participants who were randomised to and received the advanced medication reminder app intervention, 47 participants had app background data being recorded by the app developers.

#### 3.3.1. Medication Data

For the medication data, 42 of the 47 participants entered at least one medication in the app with a total of 294 medications, including both cardiovascular and non-cardiovascular medications. The median number of medications entered per patient was 5.0 (IQR 4.0), ranging from one to 15 medications. In terms of type of medications, 59.2% (174/294) of the medications entered in the apps belonged to one of the four main classes of cardiovascular medications recommended to patients with CHD, which include (1) anti-platelets, (2) angiotensin converting enzyme inhibitors (ACEi) or angiotensin receptor blockers (ARB), (3) beta-blockers, and (4) statins (Table 6). The other types of medications were hypoglycemic agents, other cardiovascular medications and non-cardiovascular medications. The overall median percentage of adherence measured in the app for all medications was 81.9% (IQR 43.1). Comparing the different medication classes, the higher percentage of adherence was for aspirin and the worst one was for beta-blockers, as shown in Table 6.

#### 3.3.2. Clinical Measurement Data

For the clinical measurement data, 40 of the 47 participants entered at least one clinical measurement in the app with a total of 262 clinical measurement data points. The median number of clinical measurement data points per patient was 2.0 (IQR 1.0), which ranged from one to 115 measurements. Only 10% (4/40) of patients had at least 15 clinical measurement data points during the median time of follow-up of 3.5 months, characterising, on average, at least one measurement per week. The main types of clinical measurements entered in the app were BP, glucose and weight measurements, accounting for 36.4% (95/262), 36.0% (94/262), and 18.8% (49/262) of the measurements, respectively. The other types of clinical measurements included HDL and LDL-cholesterol, triglycerides, INR, temperature and oxygen saturation, with less than 2% of the measurements each. Thirty-seven of the 47 participants entered at least one BP measurement, ranging from one to 18 BP entries per patient. Analysing the BP data entered in the app, the mean systolic BP of these participants was 124.2 mmHg (SD 13.9), while the diastolic BP was 78.1 mmHg (SD 10.9).

## 4. Discussion

In this mixed-methods evaluation, we analysed different quantitative and qualitative data sources to assess the reach, acceptability, utility and engagement with the apps that were used in the MedApp-CHD study. First, in regard to the reach of the app-based intervention, we found that patients with CHD who participated in the study were more likely to be younger and males compared to those ineligible to participate, primarily those who were excluded due to lack of a suitable smartphone or English proficiency. Second, our results suggest that the apps were generally well-accepted, as participants found that it was a non-intrusive way of being reminded to take the medications. Third, in terms of utility, the participants were overall positive about the usefulness of the app as a tool to improve medication-taking behaviour. The features that were found to be the most useful ones were the medication reminders and the list of medications. The feature to record clinical measurements in the app was also found to be useful in helping them to track BP, glucose and weight levels; however, only a small number of participants regularly used this feature. Last, the study participants engaged well with the app, using it frequently as a reminder tool, finding it easy to use, engaging their family members and health professionals in the app use, and reporting that they would continue to use the app beyond study completion.

The reach and potential generalisability are important aspects to consider when developing any type of intervention. Our finding that our intervention reached more younger male adults is consistent with another app-based intervention study, in which the main reason for exclusion was also the lack of a suitable smartphone. Those that were excluded for this reason were older and more frequently females than the participants included in the study [18]. Another study has evaluated the characteristics of smartphone owners compared to non-owners and also found that younger individuals were more likely to own smartphones [19]. Currently, age and gender gaps in smartphone ownership still exist and these are limiting factors in the generalisability of an app-based intervention. However, this barrier is likely to be rapidly overcome, given the increasing numbers of smartphone ownership worldwide and a trend in reducing these age and gender gaps in recent years [20].

There were some interesting aspects of this app-based intervention that emerged in this mixed-methods evaluation. Participants reported that the apps impacted on their behaviours; the medication reminders helped them in creating a routine, not only in relation to their medication-taking behaviour, but also in a broader sense of taking care of their own health. Overall, there were no differences in the perceived utility, acceptability, and app engagement reported by participants who received the basic app when compared with those who received the advanced app, which had additional customisable and interactive features. Although there was also no significant difference in medication adherence between the basic and advanced app groups, participants in both the basic and advanced app groups reported that the ability to mark the medication doses as ‘taken’ and snooze the reminder (features that are only present in the advanced app) was an important feature to further help them to monitor their treatment adherence.

Concerning the acceptability, utility and engagement with mHealth interventions, few other studies have conducted evaluations of mHealth interventions’ acceptability and factors that influence engagement. These studies have also found that these interventions were well-accepted, and that, overall, participants were satisfied with the intervention [21,22,23,24]. Similar to our results, other mHealth interventions were helpful in promoting behavioural changes, such as increasing physical activity levels and improving diet [21,22,23,25]. In addition, family and health professional support were also found to be an important engagement factor [21,23,25]. Interestingly, participants in another study also reported that being able to track their behaviour (using a pedometer to track their physical activity) was very helpful [21], similarly to being able to mark the medication doses as ‘taken’ in the advanced app. However, none of these studies evaluated an app-based intervention, all of them evaluated text-messaging programmes.

An important strength of this study is that this mixed-methods analysis focused on evaluating the features of the apps that were used in the MedApp-CHD study, not the specific apps themselves. Therefore, practical implications can be drawn from these results. First, in terms of the patient characteristics, it seems that there should not be restrictions in recommending these apps to patients, as long as patients have a suitable smartphone and feel comfortable in using apps. Second, apps aiming to improve adherence should have the following features: the ability to record the patient’s list of medications, set reminders for daily medication-taking, snooze the reminders, and mark the medication dose as ‘taken’. Third, the clinical measurements feature seems to be more useful to hypertensive and diabetic patients, which are required to regularly measure the BP or glucose levels. Finally, the apps could be used as an instrument to measure medication adherence, particularly the advanced app feature, which provided a percentage of adherence based on the ratio of ‘taken’/scheduled doses. Although we could not compare the adherence information provided by the advanced app with adherence measured by the Morisky Medication Adherence Scale, future studies could aim to validate the app percentage of adherence as an instrument to measure adherence, comparing it with the percentage of adherence measured by MEMS (Medication Event Monitoring System), for example.

This study has several limitations. Firstly, this app-based intervention only addressed forgetfulness, which is a non-intentional component of medication non-adherence. Secondly, our findings from the feedback questionnaire responses and the participants’ perceptions expressed in the focus group discussions might have been affected by social desirability responding, where the participants tend to respond in a manner that they feel is socially appropriate, although the participants were encouraged to provide honest opinions and views about the app intervention. Thirdly, the analytical data for the basic app was not available from the software developers, so it could not be analysed. In addition, the advanced app is an app that is suitable for patients with all types of health conditions, but it was only tested in those with CHD. More research is needed to continue to explore the quantitative and qualitative evidence for the use of mobile apps in improving medication adherence across a range of chronic health conditions and elucidate whether apps can also be used to minimise the risk of intentional non-adherence, such as medication side effects or a lack of perceived need for the medication.

## 5. Conclusions

This study provides important insights regarding the features that are most useful in apps that aim to improve medication adherence. This mixed-methods evaluation suggests that, currently, young male patients with CHD are more likely to use such apps. The medication reminder apps used in the MedApp-CHD study were well accepted and useful in reminding the participants to take the medications. Participants were engaged in regularly using the apps, finding them easy to use. Future studies are needed to investigate whether such apps would also be useful in other contexts, including populations of patients with a larger female representation and other chronic health conditions.

## Figures and Tables

**Table 1:** MedApp-CHD mixed-methods evaluation plan.

Objective	Data Sources
Examine the reach and potential generalisability of the app-based intervention	Screening logs
Examine the acceptability, utility and engagement with the apps	Feedback questionnaires and focus groups
Assess engagement with the app, through evaluation of app usage patterns	Analytical data collected from the app software (advanced medication reminder app group only)

**Table 2:** Examples of questions to prompt focus group discussions.

**Usefulness of the App and Specific App Features**
Overall, what did you think of using a mobile phone app to help you with your medications and heart health?
Did you like being reminded to take your medications?
What do you think about having a list of your medications on your phone?
Did you find it useful to be able to track blood pressure and weight over time?
What did you most frequently use the app for when you were using it? Which features were most and least useful?
**Perceived benefits and potential impact of the app use**
Do you think the app changed your behaviours in any way?
Do you now have better control over taking your medications and/or your heart health in general compared with before you took part in the study?
If you found the app beneficial was that because of a specific part or feature? Or was it a general effect of the app as a whole?
**Engagement with the app**
How did you use the app during your three months in the study?
How often did you have the medication reminders? How often did you record your blood pressure and weight measurements in the app?
How easy or difficult was it to use different features of the app?

**Table 3:** Differences between excluded patients and enrolled participants.

Reason for Non-Participation	Excluded Patients	Enrolled Participants (*n* = 163)	*P*-Value
No CHD (*n* = 1576)			
Age, years, mean (SD)	52.5 (14.76)	57.9 (8.91)	<0.001
Female, N/total (%)	691/1576 (43.8)	20/163 (12.3)	<0.001
No suitable smartphone (*n* = 220)			
Age, years, mean (SD)	68.0 (10.29)	57.9 (8.91)	<0.001
Female, N/total (%)	44/220 (20.0)	20/163 (12.3)	0.045
Declined to participate (*n* = 109)			
Age, years, mean (SD)	58.3 (7.70)	57.9 (8.91)	0.654
Female, N/total (%)	11/109 (10.1)	20/163 (12.3)	0.580
Not proficient in English (*n* = 97)			
Age, years, mean (SD)	63.3 (8.81)	57.9 (8.91)	<0.001
Female, N/total (%)	27/97 (27.8)	20/163 (12.3)	0.002
Use of other medication reminder apps / alarm reminders (*n* = 27)			
Age, years, mean (SD)	55.9 (7.87)	57.9 (8.91)	0.274
Female, N/total (%)	6/27 (22.2)	20/163 (12.3)	0.163

CHD, coronary heart disease; N, number; SD, standard deviation.

**Table 4:** Questionnaire respondents and focus group participants’ characteristics.

Characteristics	Questionnaire Respondents (*n* = 98)	Focus Group Participants (*n* = 15)	*P* Value
**Demographics**			
Age, years, mean (SD)	57.9 (8.89)	57.1 (9.11)	0.764
Male, N/total (%)	84/98 (85.7)	12/15 (80.0)	0.564
Ethnicity, N/total (%)			0.127
Caucasian	51/98 (52.0)	12/15 (80.0)	
Asian / Southeast Asian	33/98 (33.7)	2/15 (13.3)	
Other	14/98 (14.3)	1/15 (6.7)	
Education, N/total (%)			0.882
<10 years	1/98 (1.0)	0/15 (0.0)	
10–12 years	16/98 (16.3)	2/15 (13.3)	
>12 years	81/98 (82.7)	13/15 (86.7)	
**Medical history**			
Previous MI, N/total (%)	66/98 (67.3)	10/15 (66.7)	0.958
Prior revascularisation, N/total (%)			
PCI	67/98 (68.4)	13/15 (86.7)	0.147
CABG	21/98 (21.4)	2/15 (13.3)	0.468
PCI + CABG	7/98 (7.1)	0/15 (0.0)	0.285
Risk factors, N/total (%)			
Hypertension	60/98 (61.2)	9/15 (60.0)	0.928
Diabetes	34/98 (34.7)	3/15 (20.0)	0.259
Dyslipidemia	69/98 (70.4)	8/15 (53.3)	0.186

CABG, coronary artery bypass graft; MI, myocardial infarction; N, number; PCI, percutaneous coronary intervention; SD, standard deviation.

**Table 5:** Quotes highlighting themes that were identified in the focus groups.

**THEME 1: Medication Reminders Were Useful**
“*The big thing I like about it…It’s my phone so it’s not like someone nagging you…you know ‘take your medications’, you know?... It’s not replacing doctors. It’s not replacing chemists. It’s not replacing people…but it’s giving you the potential for a very good monitoring tool*” (D, male, 64 years, FG 1)
“*I do really use the app and I think it’s a valuable thing*” (D, female, 68 years, FG 2)
“*So in the mornings, it’s…I’m in a routine so it doesn’t bother me but the afternoon it’s good because I come home from work, have dinner, do whatever, watching TV…I always forget to take my tablets*” (M, male, 47 years, FG 2)
“*Yeah, I think it’s definitely helped me. I, even two years of taking medication for a heart condition, I still would forget if I didn’t have something beeping at me constantly, so I think it’s helpful*” (M, male, 57 years, FG 2)
**THEME 2: List of medications was useful**
“*What I do love about it* [the app] *is I’ve got a walking inventory of everything I take*” (D, male, 64 years, FG 1)
“*When you go to the doctors and all you’ve gotta do is say ‘Here’s the app’ and it shows them every update* [on the medications list].” (D, female, 68 years, FG 2)
“*Whenever I go to emergency…then they’ll say ‘Are you on any meds?’ and I’m like ‘Yes, let me pull this* [the app] *up because I’m not gonna remember every single one’…so that’s what’s been the most helpful for me*” (P, male, 50 years, FG 2)
**THEME 3: Continue to use the app**
“*It was an app I’ll definitely keep on my phone and update if I need, if I go on a new medication*” (T, female, 61 years, FG 1)
“*I’m still using it*” (D, female, 68 years, FG 2)
**THEME 4: Recommend to family and friends**
“ *Oh, I have. I’ve suggested it* [the app] *to him* [a friend]” (D, male, 64 years, FG 1)
“*I’ve even said to other friends if you’re taking medication…it’s not a bad application to* [have]” (M, male, 57 years, FG 2)
**THEME 5: Recording clinical measurements regularly was useful**
“*I keep putting them in* [the clinical measurements in the app] *…I test myself* [for glucose levels] *8 to 10 times a day…it’s some sort of record…to try and show a chart of it*” (A, male, 64 years, FG 1)
“*I was recording my BP every day…and, you know, once a week or fortnight recording my weight*” (M, male, 57 years, FG 2)

BP, blood pressure; FG, focus group.

**Table 6:** Types of medications entered in the app and percentages of adherence.

Medication Class	Frequency of Medication Class N/total Number of Medications (%)	Percentage of Adherence Median (IQR)
Aspirin	44/294 (15.0)	85.3 (32.9)
Other anti-platelets	28/294 (9.5)	78.5 (38.0)
ACE inhibitors/ARBs	29/294 (9.9)	84.0 (38.6)
Beta-blockers	25/294 (8.5)	74.6 (42.1)
Statins	48/294 (16.3)	84.0 (37.7)
Hypoglycemic agents	34/294 (11.6)	76.9 (34.7)
Other cardiovascular medications	32/294 (10.9)	81.7 (47.7)
Other non-cardiovascular medications	54/294 (18.4)	84.3 (41.8)

ACE, angiotensin converting enzyme; ARB, angiotensin receptor blocker; IQR, interquartile range; N, number.
